# Asymmetric air-sea heat flux response and ocean impact to synoptic-scale atmospheric disturbances observed at JKEO and KEO buoys

**DOI:** 10.1038/s41598-020-80665-8

**Published:** 2021-01-11

**Authors:** Hiroyuki Tomita, Meghan F. Cronin, Shun Ohishi

**Affiliations:** 1 Institute for Space-Earth Environmental Research (ISEE), Nagoya University, Nagoya, 464-8601 Japan; 2 Pacific Marine Environmental Laboratory, NOAA, Seattle, WA USA; 3 Data Assimilation Research Team, RIKEN Center for Computational Science, Kobe, Japan

**Keywords:** Ocean sciences, Physical oceanography

## Abstract

This study aims to identify patterns of surface heat fluxes, and corresponding surface ocean responses, associated with synoptic-scale atmospheric events and their modulation on seasonal time scales. In particular, northerly and southerly wind events associated with atmospheric disturbances were analyzed using high-temporal resolution time-series data from two moored buoys (JKEO: 2007–2010 and KEO: 2004–2019) north and south of the Kuroshio Extension current. Although each synoptic-scale wind event generally impacted both sites, the composite surface heat flux was larger at the northern site, especially for northerly events. Both types of wind events were observed throughout the year, with a minimum during June-July–August. Northerly wind events tended to be accompanied by lowered air-temperature, while southerly events tended to have elevated air-temperature relative to the previous three days. The resulting anomalous surface heat loss was asymmetric, with larger changes in northerly events compared to the southerly events. A large and significant ocean response of − 0.28 to − 0.46 K (p-value < 0.05) in SST was confirmed only for northerly events in spring–summer at the northern site, while smaller changes were found at the southern site. The results of this study suggest that sub-monthly air-sea interactions may affect seasonal variability and potentially climate change over longer timescales.

## Introduction

**Figure 1:**
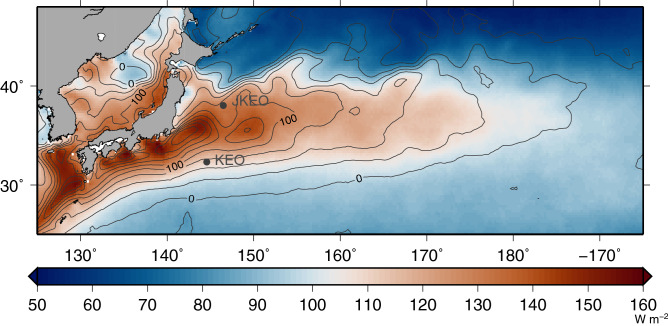
Location of the JKEO and KEO buoys in the Kuroshio Extension region. Colours show the magnitude of variation of the net heat flux at the synoptic time scale, defined as the standard deviation of high-pass-filtered satellite-derived air–sea heat flux (J-OFURO3 daily mean data)^8^. The high-pass filter was based on 60 days running mean. Contours represent the long-term mean of the J-OFURO3 surface net heat flux (1988–2017). Positive values imply heat flux from the ocean to the atmosphere. Units are in W/m^2^. The Generic Mapping Tools (version 5, https://www.generic-mapping-tools.org) was used to create this figure.

Western boundary currents are strong, warm currents that transport heat from the tropics to higher latitudes, where the warm surface water makes contact with cool and dry air, generating large surface heat fluxes during wintertime that warm the atmospheric boundary layer. As a consequence, western boundary currents, as they extend into the ocean interior, are “hot spots” for air-sea interaction. The Kuroshio Extension (KE) region is such a "hot spot" for the North Pacific (Fig. 1). Surface heat release from the ocean to the atmosphere dominates in winter. By contrast, weak heat input from the atmosphere to the ocean occurs in spring–summer. At shorter (i.e., sub-monthly) time scales, surface heat fluxes have large variations associated with synoptic-scale atmospheric disturbances^1,2^. The focus of this study is identifying surface heat flux response associated with synoptic-scale atmospheric events at a time scale with several days. In this study, we analyze the characteristics and patterns of surface heat flux associated with these synoptic-scale atmospheric disturbances, their seasonality, and the ocean responses to these atmospheric disturbances.

The characteristics described from a large number of atmospheric events provide information as a baseline against which responses to individual atmospheric events can be compared. Because these events are irregularly distributed throughout the year and can have asymmetric and complex responses, understanding these patterns is critical for understanding seasonal and longer variations. More recent studies have pointed out the importance of understanding synoptic-scale wind changes and corresponding changes in surface heat fluxes for an understanding of the climatological mean field of surface heat flux^3,4^. Moreover, observational evidence of sub-monthly fluxes and their ocean response will be useful for validating satellite observations and coupled atmospheric-ocean numerical weather prediction models, whose reliability is not yet completely established^5^.

Using in situ observations from the early 1990’s off the southern coast of Japan, Qiu et al.^1^ showed that the surface flux associated with synoptic-scale atmospheric conditions had a dominant time scale of 3–14 days. As shown in Fig. 1, since 2004, a moored buoy, referred to as the Kuroshio Extension Observatory (KEO), has monitored air-sea interaction south of the KE. Likewise, from 2007 to 2010 a moored buoy, referred to as the JAMSTEC-KEO (JKEO) was stationed north of the KE^6^. These high-temporal resolution moored buoy observations make it possible to study in detail conditions responsible for the surface heat flux variations. For example, Bond and Cronin^2^ studied the weather patterns that result in extremely large surface heat fluxes at the KEO buoy location. Furthermore, Konda et al.^7^ analyzed the spatial differences in flux associated with synoptic-scale atmospheric variations using in situ observations from the two buoys. Both of these early studies were based upon less than 2 years of data. Neither study evaluated composite surface meteorological and oceanic time series associated with these atmospheric disturbances. A revisit of these studies, with a more in-depth analysis of the surface atmospheric characteristics and oceanic response to these synoptic events, is thus warranted. To address this, the present study focuses on the local surface heat flux and SST responses to synoptic-scale atmospheric disturbances. Furthermore, we consider the rectification of this sub-monthly variability into the seasonal cycle.

## Results

### Regional view

Before examining the buoy observations, we briefly review the magnitude and extent of atmospheric synoptic-scale variability over the KE. Figure 1 shows the distribution of the long-term mean surface net heat flux (contour) derived from satellite observations^8^ and the standard deviation (colour) using a high-pass filter based on two-month running mean. While net heat release from the ocean to the atmosphere dominates over the KE region, it has a complex spatial pattern due to the influence of the Kuroshio Current, and its oceanic front and eddies, as they interact with the synoptic weather events. In particular, the amplitude of the synoptic-scale variation in surface net flux is large at locations where the average heat flux is large. This is confirmed at the buoy locations and by the buoy observations: At the buoy locations (JKEO and KEO; Fig. 1), the total average heat flux was 83.2 and 68.9 W/m^2^, respectively, and the standard deviations of the high-pass-filtered variations were 130.2 and 111.3 W/m^2^, respectively. The net heat release and amplitude of the high-pass-filtered variation at JKEO were slightly higher as compared to KEO.

### Typical atmospheric synoptic time scale flux variation

**Figure 2:**
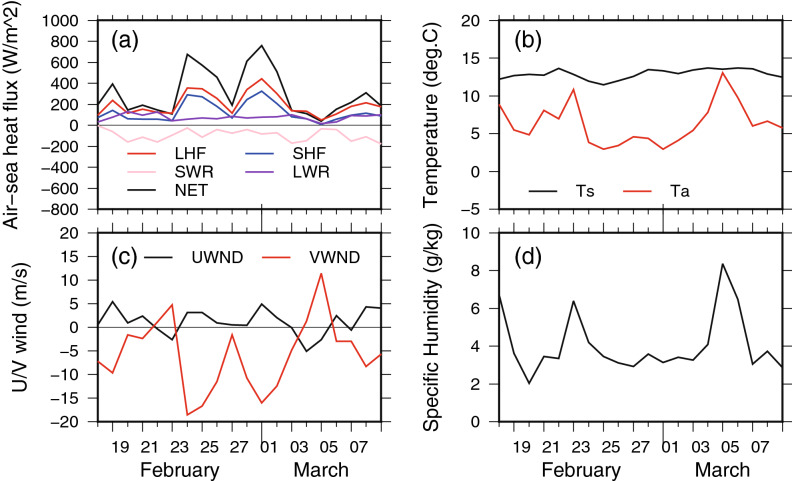
Typical daily time series of surface heat fluxes and related physical parameters observed at JKEO from 18 February to 8 March 2007 (30 days). LHF, SHF, SWR, LWR, and NET indicate the surface latent heat flux, sensible heat flux, net shortwave radiation, net longwave radiation, and net heat flux, respectively. T_s_, T_a_, UWND, and VWND are the sea surface temperature, air temperature, zonal components, and meridional components of surface winds, respectively. Full time series of JKEO and KEO are shown in Supplementary Figs. S1 and S2, respectively.

As an example, Fig. 2 shows the daily time series of surface heat fluxes observed at JKEO for 30 days from 18 February 2007 to 8 March 2007. The surface winds changed markedly over several days during this period, showing both northerly and southerly peaks. Atmospheric temperature and specific humidity varied in line with the wind peaks, and the surface heat flux also changed. Such changes in surface winds with northerly/southerly wind peaks are generally seen throughout the year at both buoys (see Supplementary Figs. S1 and S2). The co-variabilities were also confirmed for the entire data period as the correlation coefficients between high-passed filtered time-series of the meridional component and atmospheric temperature, specific humidity, and surface net heat flux were 0.74, 0.61, and − 0.69, respectively.

### Northerly/southerly event composite at the JKEO buoy

To quantitatively investigate variations in the surface heat flux on an atmospheric synoptic time scale, we constructed composite timeseries of surface heat fluxes and associated variables based on southerly/northerly wind peaks over the entire JKEO period (17/02/07–15/06/10) (see “Data and methods”).

**Figure 3:**
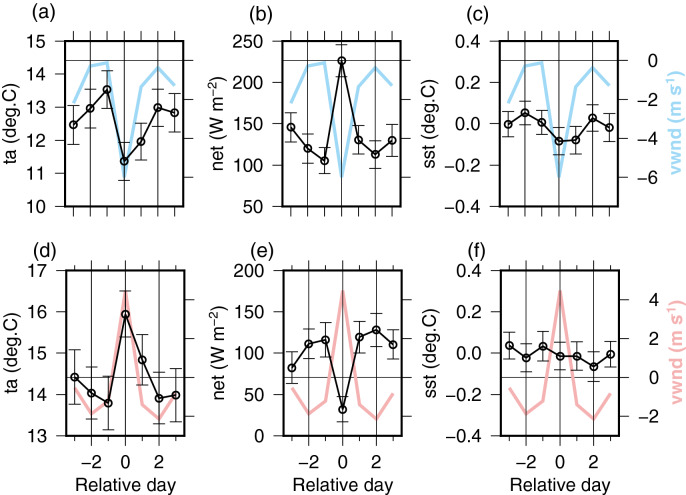
Northerly (**a**–**c**) and southerly (**d**–**f**) wind composite time series at JKEO: (**a**,**d**) surface air temperature (ta), (**b**,**e**) surface net heat flux, (**c**,**f**) high-pass filtered sea surface temperature (sst). A second order Butterworth high-pass filter was used in this analysis. The horizonal axis indicates the relative day from the peak (0). The error bars indicate standard error. The red and blue lines indicate the meridional component of surface winds (vwnd) for northerly and southerly wind events, respectively. The figures for other components of flux and related variables are shown in Supplementary Figs. S4 and S5.

First, the northerly and southerly wind composites (Fig. 3) and corresponding weather patterns were evaluated (Supplementary Fig. S3). At the peak of northerly wind, the distribution of sea level pressure (SLP) shows high pressure over the Japan Sea and low pressure in the area of east of Japan. On the other hand, at the peak of the southerly wind, the distribution of SLP has almost the opposite pattern, showing low pressure over the Japan Sea and high pressure over the area east of Japan. These results suggest a close relationship between prevailing atmospheric synoptic-scale disturbances and surface wind direction. Typically, these weather disturbances, associated with northerly and southerly wind events, follow the North Pacific storm track, moving rapidly from west to east^9^.

Figures 3a–c show the composite result for the northerly events at JKEO. Surface wind changes averaged − 5.9 m/s from the previous day to the peak and marked air temperature cooling (approximately − 2.2 °C) were apparent; air temperatures reach to the minimum and then was restored within two days after the peak. The estimated maximum SST cooling (− 0.14 °C) was considerably smaller than that of the surface air temperature and not significant.

These changes at JKEO during the northerly wind event resulted in a peak surface net heat loss (Fig. 3b) with a change of 121 W/m^2^ from the previous day, due primarily to increases in latent and sensible heat fluxes and secondarily to the contributions of radiations (see the later subsection, “Anatomy of surface heat flux response” for details). The change from previous days to the peak is significant with p-value (< 0.0001) from t-test.

The JKEO composite of the southerly wind event also showed clear changes in air temperature and changes in surface net flux (Fig. 3d–f). The north–south wind varied by a maximum of 6.6 m/s from a weak northerly to an average southerly peak of 4.5 m/s, with air temperature increasing during the southerly event by approximately 2.2 °C. A peak in surface net heat flux was also evident; however, the change of − 95 W/m^2^ was significantly smaller compared to the composite results for the northerly wind event with p-value (< 0.01) from t-test. However, the change is statistically significant with p-value (< 0.0004) from t-test. No clear change in SST was detected for the southerly wind event.

### Comparison with results at the KEO buoy

**Figure 4:**
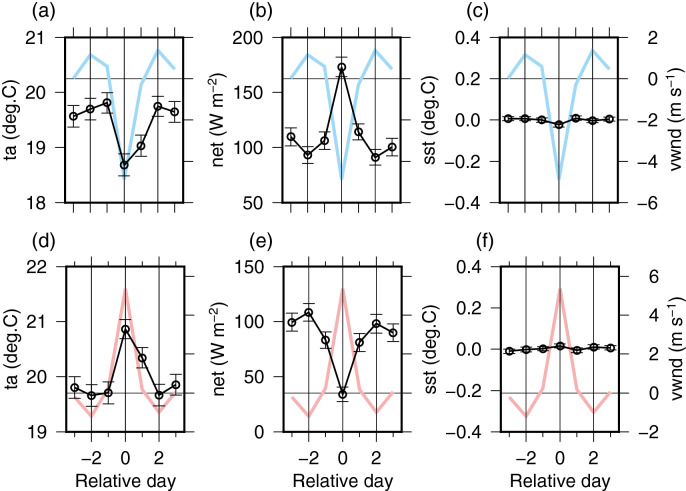
Same as Fig. 3, but for KEO. The figures for other components of flux and the related variables are shown in Supplementary Fig. S6 and S7.

A comparative analysis using the long time series (16/06/04–25/09/19) from KEO, moored approximately 600 km south of JKEO (Fig. 1 and Supplementary Tables S1 and S2), revealed similar changes in air temperature and surface heat flux associated with northerly/southerly wind events (Fig. 4). The maximum changes in net heat flux for northerly and southerly wind events are 81.7 and − 74.7 W/m^2^, respectively. At KEO, the heat release tends to be slightly smaller compared to JKEO; however, the pattern of variation is very similar. The correlation coefficient and root-mean-squared difference between KEO and JKEO is 0.91 and 19.1 W/m^2^, respectively. If the difference in standard deviation calculated using data extracted for each wind event is used as an indicator of the amplitude of flux response to the atmospheric synoptic time scale fluctuations, the difference is 11.9 W/m^2^, which is slightly larger for JKEO. These results show that the surface flux response to synoptic-scale atmospheric variations is virtually the same at JKEO and KEO; however, JKEO responds with a slightly larger amplitude.

The characteristics of the ocean response at KEO differed from those observed at JKEO. A decrease in SST of 0.14 °C was observed at JKEO during the northerly wind event (Fig. 3c), while the decrease at KEO was quite small (approximately − 0.03 °C), but statistically significant (p-value < 0.04) from t-test (Fig. 4c).

### Anatomy of surface heat flux response

As shown in Figs. 3 and 4, much of the surface net heat flux fluctuations associated with northerly and southerly wind events can be attributed to changes in air temperature; but here, other factors (changes in radiations, scalar wind speed, and water temperature) are also considered. The extent of these contributions will be evaluated quantitatively by analyzing a linearized bulk formula (see “Data and methods”).

**Figure 5:**
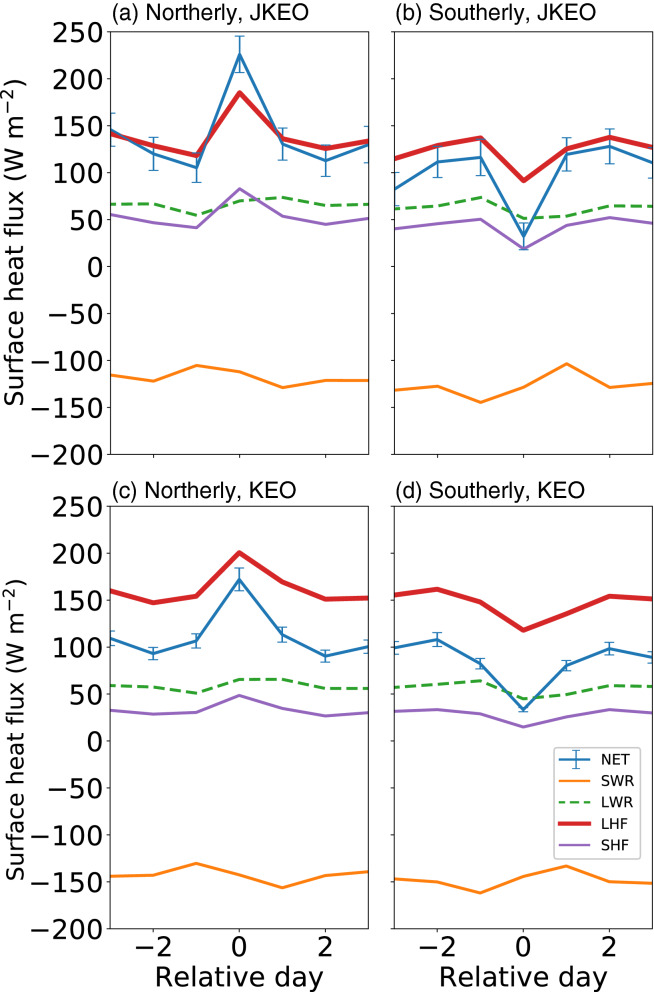
Surface heat flux composition. (**a**) and (**b**) are northerly and southerly composites at JKEO, respectively. (**c**) and (**d**) are northerly and southerly composites at KEO, respectively. NET, SWR, LWR, LHF, and SHF indicate the surface net heat flux, net shortwave radiation, net longwave radiation, surface latent heat flux, and sensible heat flux, respectively. The error bars indicate standard error for NET.

First, the composite time series of each surface heat flux component is computed. As shown in Fig. 5, the surface net heat flux fluctuations are dominated by LHF, followed by SHF, regardless of whether it is a northerly or southerly wind events. In particular, the fluctuation of LHF and SHF accounts for more than 80% of the net surface heat flux anomaly. A weak change in the radiative flux was also observed due to clouds, which caused increased cooling due to net long-wave radiation and decreased warming due to short-wave radiation. These counteracting radiative effects resulted in a uniform offset of approximately 20 W/m^2^; contributing less than 20% to the net surface heat flux anomaly. This radiative feature was common to both JKEO and KEO buoys. According to these results, most of the surface net heat flux fluctuations associated with northerly/southerly wind events are due to fluctuations in turbulent heat flux (THF = LHF + SHF).

**Figure 6:**
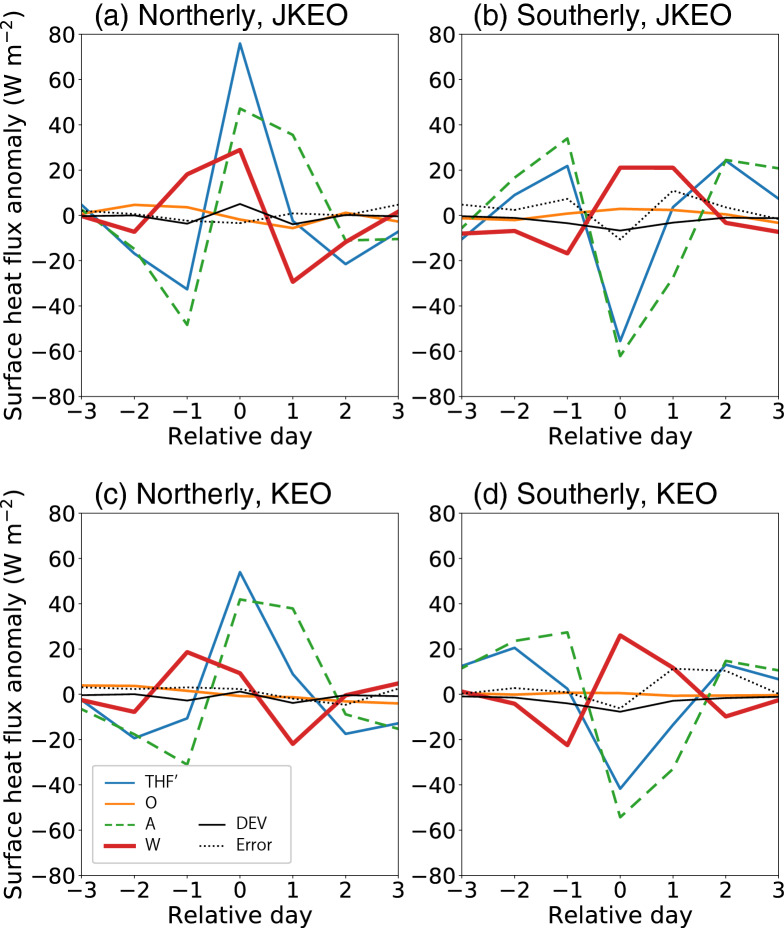
Surface turbulent heat flux variation and its contributing factors. (**a**) and (**b**) are northerly and southerly composites at JKEO, respectively. (**c**) and (**d**) are northerly and southerly composites at KEO, respectively. THF’ indicates an anomaly from the temporal mean during the 7-day composite. O, A, W, DEV, and error are contributions from changes in the ocean, atmosphere, winds, their perturbations, and error. See “Data and methods” for details.

Second, since THF is a function of water temperature, air temperature, humidity, scalar wind speed, etc., here, the THF fluctuation is analysed in further detail by evaluating each term of the linearized bulk equation. As shown in Fig. 6, the fluctuation mechanism of THF is very similar between JKEO and KEO. Overall, the contribution of atmosphere (A), i.e. contribution of temperature and specific humidity variations is the main factor of THF variation, and the contribution of wind speed variation (W) is secondly large. Interestingly, the fluctuation factor is different between northerly and southerly wind events as follows.

At the northerly wind peak, approximately 50% of the fluctuation of THF is explained by variability in the surface air temperature and humidity, i.e., the contribution of A, and about 20% is explained by scalar wind speed changes, i.e. the contribution of W. The contribution of the ocean (O) is fairly small, and the water temperature drops slightly, contributing to reducing the fluctuation of THF with a contribution of about − 1%. At the southerly wind peak, there is almost the same or slightly larger contribution of A to the THF, while the contribution of W acts to cancel the contribution of A. The differences in the mechanism of THF fluctuation between the northerly and southerly wind events revealed here are thought to have caused the asymmetry of the surface heat flux response.

### Seasonal dependence

**Figure 7:**
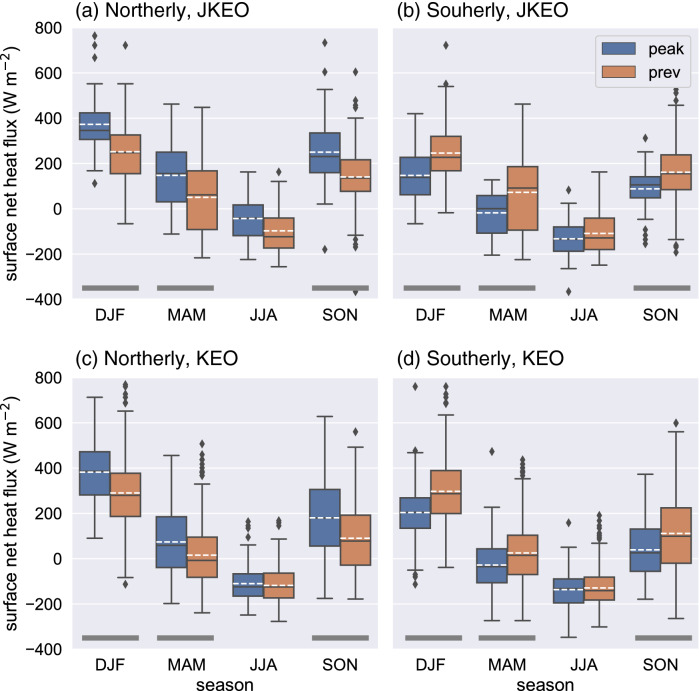
Seasonality of surface net heat flux response to the (**a**,**c**) northerly and (**b**,**d**) southerly wind events. The changes in the surface net heat flux between the peaks and its previous 3 days are shown as box plots for the two buoy sites: (**a**,**b**) JKEO and (**c**,**d**) KEO. Seasons are defined as four different periods: December, January, and February (DJF); March, April, and May (MAM); June, July, and August (JJA); and September, October, and November (SON). Significant differences (99%) between the peaks and its previous 3 days are indicated by a thick black bar at the bottom. The box plots were depicted with the minimum, the maximum, the median, and the first and third quartiles. The white dashed lines and black diamonds are showing means and outliers.

The results may be seasonally dependent because of seasonal differences in a number of occurrences of the wind events, their strength, and atmospheric and oceanic conditions. In order to check seasonal dependence of surface heat flux response to northerly/southerly events, the changes were compared among composites calculated in each season. Figure 7 shows that the significant changes in surface net heat flux relative to the previous 3-days at JKEO and KEO. At JKEO, significant changes were found in boreal winter, spring, and autumn seasons with the p-values from the t-test, but in boreal summer the changes were quite small and insignificant. Likewise, the changes in surface net heat flux at KEO were significant in boreal winter, spring, and autumn seasons but the values were slightly smaller than JKEO. All values for the statistical t-test and p-values are also listed in Supplementary Table S3.

**Figure 8:**
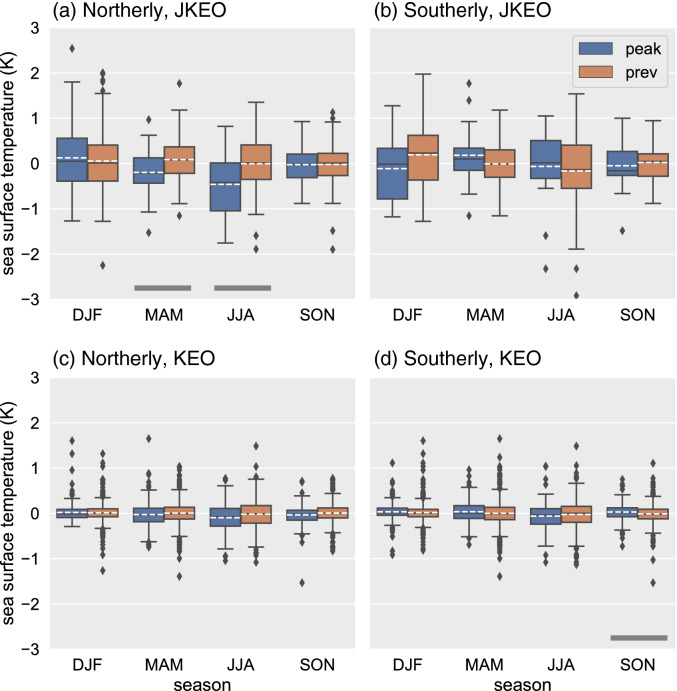
Seasonality of the sea surface temperature response to the (**a**,**c**) northerly and (**b**,**d**) southerly wind events. Significant differences (95%) are indicated as thick black bars at the bottom. Other configurations are same as Fig. 7.

Likewise the oceanic response had distinct seasonal differences. Figure 8 shows the significant changes in SST relative to the previous 3 days at JKEO and KEO. Changes at JKEO were found in spring and summer seasons for the northerly wind event cases with the P-values from t-test of 95% confidence level. On the other hand, there were no significant changes for the southerly event at JKEO. At KEO, a significant ocean response was confirmed only for southerly events in autumn. All values for the statistical t-test and p-values are also listed in Supplementary Table S4. Similar results were confirmed by simple correlation analysis. Supplementary Table S5 shows that at JKEO, the correlation coefficient tends to be significant and higher in spring and summer, while at KEO, the correlation is lower. In this correlation analysis, it is interesting to note a significant correlation even in the southerly wind event, although the above composite analysis did not show a significant change in SST.

### Reason for the oceanic response

First, we focus on the northerly wind event at JKEO because the surface net heat flux changed markedly, and a significant and large ocean response was observed with surface temperature changes in boreal spring season (see Fig. 8 and Supplementary Table S4). The change in water temperature in the ocean mixed layer (~ SST) at JKEO can be expressed by Eq. (1). The observed SST change associated with the northerly wind event was approximately − 0.3 °C/day (Supplementary Table S4) and surface net heat flux peaked at approximately 147 W/m^2^ (Supplementary Table S3). The thickness of the ocean mixed layer was approximately 30 m. Consequently, the change in mixed layer temperature due to surface net heat flux is calculated to be 0.11 °C/day, explaining approximately 37% of the total SST change. The standard errors of the surface net heat flux and the thickness of the ocean mixed layer were estimated as 20 W m^−2^ and 5 m, respectively. These correspond to temperature change errors of ~ 0.03 °C/day.

While larger change in surface net heat flux was observed in boreal autumn and winter, the ocean mixed layer in these seasons is approximately three times thicker than in spring. Consequently, the change in water temperature caused by the surface net heat flux is < 0.1 °C/day, considerably smaller than the estimate in boreal spring. At KEO, the averaged ocean mixed layer is 140 m and substantially thicker than that of JKEO. Consequently, the oceanic response to the net heat flux anomaly is quite weak.

## Summary and discussion

The purpose of this study was to examine the surface heat flux response to synoptic-scale atmospheric variations and the corresponding oceanic response at two long-term surface buoy stations, the JKEO buoy located north of the KE current and the KEO buoy located to its south. Using KEO net surface heat flux time series and atmospheric reanalysis data, Bond and Cronin^2^ showed that large (> 1σ), positive anomalies in the net surface ocean heat loss values were associated with weather patterns with northerly winds at the buoy site; while large, negative anomalies were associated with southerly winds at the buoy site. Thus for this analysis, we used these long buoy time-series to create composite time series for northerly and southerly wind events.

Overall, roughly equal or slightly more events occur at JKEO as at KEO: the occurrence per year frequency was 84.2 (67.3) northerly events at JKEO (KEO) and 72.7 (69.4) southerly events at JKEO (KEO), with both types of wind events observed in all seasons (Supplementary Tables S1, S3, and S4). The results are consistent with these single-year winter patterns found during the previous studies^2,7^: the flux response shows a larger net ocean surface heat loss in the northerly wind events, than southerly wind events, and the larger heat loss was found at the JKEO site north of the KE, than at the KEO site. Our analysis shows that these results extend to other seasons (only JJA was not significant), and was robust over several years at JKEO and KEO.

Interestingly, an asymmetric surface heat flux response showing larger changes in northerly wind events compared to the southerly events was found. The asymmetric response contributes to the mean state of surface heat flux at seasonal or longer time scales (see also Supplementary Fig. S8). In particular, as noted in Fig. 1, where the variability is large, the mean net surface heat flux is large. In order to quantitatively evaluate the rectification effect of the asymmetric flux response, we compared the averaged values obtained from the original time series, which includes the observed asymmetric response, with the values calculated from the time series that has artificially removed the asymmetric features. This artificial modification was made by simply multiplying by a factor to reduce the value at northerly wind peak. The factors at JKEO and KEO are 0.7 and 0.4, respectively. The results are summarized in Supplementary Table S6. At both JKEO and KEO buoys, the averaged value calculated from the time series with artificially removed asymmetric features is 17–20 W/m^2^ smaller than the averaged value calculated from the observed original time series. From this result, it is estimated that the characteristics of the asymmetric flux response have the effect of increasing the averaged value of surface net heat flux by 16–30% over the entire period. The table S6 also shows that the characteristics of northerly wind events, which account for only 18–22% of the total days, dominate the record-averaged value.

Our results are consistent with a recent study^4^ that reports the importance of surface wind direction on the synoptic time scale for the long-term mean field of surface turbulent heat flux. In addition, their study suggested the necessity to consider sub-weekly time scales to describe the valid surface heat exchange. The same thing can be confirmed from the investigation of the time scale dependence of the surface heat flux response (Supplementary Fig. S9). The flux response and asymmetric feature are quickly reduced from daily to weekly time window. The daily or less time resolutions are indispensable to investigate the response.

Moreover, our analysis also focused on the corresponding synoptic variability in the surface meteorological measurements, the surface heat flux components (including the radiative components), and the SST response. The asymmetry in the net surface heat loss associated with northerly and southerly events also led to an asymmetric ocean response that is compounded by the differences in the ocean mixed layer. As discussed by Tozuka et al.^10^, because the ocean mixed layer is deep on the warm side of the KE, SST is relatively insensitive there to surface heat fluxes. As a consequence, the SST response was significantly larger at JKEO than at KEO, although even at JKEO, the mean SST response was < 0.5 K, and similar to the previous results found off Shikoku, Japan^1^. Because these synoptic events are associated with both convective mixing and wind stirring within the ocean^11^, it is likely that the heat anomaly is mixed relatively deep and results in only a weak surface temperature change. Dynamical features of the ocean itself, such as water temperature fluctuations influenced by mesoscale eddies and meanders, are frequently observed and appear to be more effective at causing large SST variations. Nevertheless, the tendency for these atmospheric disturbances to preferentially cause cooling on the north (i.e., cool) side of the KE indicates that these disturbances help maintain the intensity of the SST front. To better understand how deep the ocean response extends, sub-surface ocean data must be analysed. Ideally, these continuous subsurface data, collocated with the surface flux data, would have higher vertical resolution than is currently available.

Availability of long, high-resolution high-quality air-sea flux data is critical for analyses such as performed here. For this study we used data from two buoys (JKEO and KEO), which are more accurate than satellite-based products, particularly at the short-scales targeted in this study^8^. On the other hand, buoy time series are often too short to capture decadal variability such as occurs in the Kuroshio and Oyashio current systems^12,13^. Analysis of the KEO buoy, which was initiated in 2004, shows a significant ocean response (− 0.03 K, p-value = 0.02) during the stable period of the KE, while the ocean response was unclear during the unstable period (2006–2009). During KE unstable periods, influence from the KE can lead to large fluctuations in SST in the western Kuroshio-Oyashio confluence region where JKEO is located^14^. The observation period of JKEO (2007–2010) is shorter than KEO and for most of its period corresponds to the unstable period of KE. Despite the strong influence of KE with its large fluctuations in SST, the present study revealed clear and significant ocean responses (− 0.28 to − 0.46 K, p-value < 0.05) in spring–summer at JKEO. Moreover, since the above analysis of KEO data showed a clearer ocean response during the KE stable period, it can be expected that there will be a larger and clearer ocean response during the KE stable period. A more detailed analysis of the decadal scale is the next step.

While satellite or numerical weather prediction models can potentially be used to provide information about the spatial response to these synoptic atmospheric events, a careful assessment of their uncertainties and biases must first be performed. Confirming simulations from the latest coupled atmosphere–ocean model is also vital for obtaining better predictability. This study provides composites of the air-sea heat flux response to synoptic-scale southerly and northerly wind events and their impact on the ocean surface. In addition to providing baseline observations for model assessments, the results contribute to better understanding of the relationship between SST and synoptic-scale surface heat fluxes.

## Data and methods

### Observational data

Time series data of surface meteorological parameters observed by two moored buoys in the KE (KEO and JKEO) were used (Fig. 1). Observations of SST, air temperature, relative humidity, wind speed and direction were recorded every 10 min. Shortwave and longwave radiation were recorded every two minutes and these were averaged for every 10 min. From these observed data, high-temporal resolution (10 min interval) air-sea turbulent (latent and sensible) heat flux was calculated at each buoy using the COARE 3.0 flux algorithm^15^. Net longwave and shortwave radiation were calculated using the method described in^16^. Surface net heat flux was calculated as the sum of turbulent heat fluxes and net radiation. Daily mean air-sea fluxes and surface parameters were calculated from the 10 min interval data. Temporal coverage differed at each buoy and small data gaps occurred due to sensor damage, buoy drifting, and technical problems in data processing. Notwithstanding, 542 (3324) days of data were available for JKEO (KEO). The temporal coverage and volume of data for each buoy are summarised in Supplementary Table S1. The average and standard deviation for observed variables and calculated surface heat fluxes are also summarised in Supplementary Table S2. A full time series of daily mean air-sea heat fluxes at JKEO during 2007–2010 (Supplementary Fig. S1) shows that seasonal variations in air-sea heat fluxes, surface metrological parameters, and SST are obvious. Heat flux variations on time scales of several days are also apparent. Same features were shown in a full time series at KEO during 2004–2019 (Supplementary Fig. S2).

Both JKEO and KEO mount sensors for conductivity and temperature (CT) or conductivity, temperature, and depth (CTD) on the sub-surface mooring line to monitor hourly water temperature, salinity, and pressure. While these data can be used to estimate the thickness of the ocean mixed layer defined as the depth at the density increases by 0.03 kg m^−3^ or temperature decreases 0.2 K (if density are not available), CT/CTD sensors were sparsely arranged in the water column (22–33 sensors to a depth of 600 m). Hence, these mixed layer depth estimates are used primarily to discuss difference between the JKEO and KEO mixed layers.

The response of SST to synoptic-scale surface flux was much weaker than the change in air temperature. Therefore, a high-pass filter was applied to oceanic data to extract only the high-frequency fluctuations. The high-pass filter was a second-order Butterworth filter with a power of 1/√2 at a cut off period of 14 days. To minimise end effects of the filter, 50 zero-patting was applied at both data ends. Moreover, to avoid adverse end effects, data for the first and last 30 days were excluded from the analysis.

### Wind direction composite

To investigate surface heat flux variations related to synoptic-scale atmospheric disturbances, two kinds of composites were calculated depending on the wind direction (northerly and southerly winds). The peaks of northerly and southerly winds were sought for a three day time series segment. Peaks were included only if the absolute value of each peak was larger than the climatological value, thereby excluding the impact of small energy perturbations. A total of 125 (613) northerly and 108 (632) southerly peaks were detected at JKEO (KEO). For the all detected peaks, a daily composite was calculated for seven days, centred on the peak day (the peak day ± 3 days). Finally, the seven day composite time series were compiled for the northerly and southerly wind peaks at each buoy.

### Ancillary data

To highlight the spatial patterns of the composites, daily mean atmospheric reanalysis data from NCEP/NCAR^17^ and a satellite-derived surface heat flux product, J-OFURO3^8^, were used in Supplementary Fig. S3 and Fig. 1, respectively. Although the spatial resolution of the NCEP/NCAR reanalysis is relatively low (T62; approximately 2°), it is sufficient to resolve synoptic-scale atmospheric features in variables such as sea level pressure and surface wind. Conversely, the spatial resolution of J-OFURO3 is 0.25º; hence, these data are preferred to resolve fine scale features of surface heat flux in terms of local air-sea interaction over oceanic fronts or meso-scale eddies with spatial scales < 100 km.

### Heat budget in the ocean mixed layer

To quantify the impact of variations in surface flux corresponding to atmospheric synoptic disturbance on the ocean mixed layer, the heat budget in the ocean mixed layer was analysed. The time change of the water temperature in the ocean mixed layer, ∂T/∂t, can be expressed by Eq. (1):

1 $$\frac{\partial T}{{\partial t}} = \frac{Q}{{\rho C_{p} H}} + other\; process$$

where *T, Q, ρ, C*_*p*_*,* and* H* are temperature in the ocean mixed layer, surface net heat flux, density of water, specific heat capacity of water, and thickness of ocean mixed layer, respectively. The first term on the right side is the effect of surface heat flux, and the rest is the effect of other processes (i.e. entrainment, oceanic advection and diffusion processes). In this study, the ∂T/∂t and surface heat flux terms were estimated from buoy observation data, and we assumed that T was approximately equal to SST. The oceanic process term was estimated as a residual.

### Linearized bulk formula for surface turbulent heat fluxes

To quantify factors of surface turbulent heat flux variations, analysis using linearized bulk formulae^18^ was adopted in this study. The formulae for surface latent and sensible heat fluxes are expressed as follows:

2 $$LHF^{\prime} = LHF - \overline{LHF} = \rho_{a} LC_{E} \left\{ {\overline{W}Q_{s }^{^{\prime}} - \overline{W}Q_{a }^{^{\prime}} + W^{\prime}\left( {\overline{{Q_{s} }} - \overline{{Q_{a} }} } \right) + \left[ {W^{\prime}\left( {Q_{s }^{^{\prime}} - Q_{a }^{^{\prime}} } \right) - \overline{{W^{\prime}\left( {Q_{s }^{^{\prime}} - Q_{a }^{^{\prime}} } \right)}} } \right]} \right\}$$

3 $$SHF^{\prime} = SHF - \overline{SHF} = \rho_{a} C_{p} C_{H} \left\{ {\overline{W}T_{s }^{^{\prime}} - \overline{W}T_{a }^{^{\prime}} + W^{\prime}\left( {\overline{{T_{s} }} - \overline{{T_{a} }} } \right) + \left[ {W^{\prime}\left( {T_{s }^{^{\prime}} - T_{a }^{^{\prime}} } \right) - \overline{{W^{\prime}\left( {T_{s }^{^{\prime}} - T_{a }^{^{\prime}} } \right)}} } \right]} \right\}$$

where *LHF, SHF, ρ*_*a,*_* L, C*_*p*_*, C*_*E*_*, C*_*H*_*, W, Q*_*s*_*, Q*_*a*_*, T*_*s*_*,* and *T*_*a*_ are surface latent heat flux, sensible heat flux, air density, latent heat of water, heat capacity, bulk transfer coefficients for latent and sensible heat flux, surface scalar wind speed, saturated specific humidity at surface, specific humidity, sea surface temperature, and air temperature, respectively. $\overline{V}$ and $V^{\prime}$ imply a temporal mean and deviation from the mean for a variable *V*. Turbulent heat flux (THF) is defined as the sum of LHF and SHF, and its deviation is expressed as follows:

4 $$THF^{\prime} = LHF^{\prime} + SHF^{\prime} = O + A + W + DEV + Error{ }$$

where $O = \rho_{a} LC_{E} \overline{W}Q_{s}^{^{\prime}} + \rho_{a} C_{p} C_{H} \overline{W}T_{s}^{^{\prime}}$ is a contribution of changes in ocean, i.e., saturated specific humidity and sea surface temperature; $A = \rho_{a} LC_{E} \overline{W}Q_{a}^{^{\prime}} + \rho_{a} C_{p} C_{H} \overline{W}T_{a}^{^{\prime}}$ is a contribution of changes in atmospheric humidity and air temperature; $W = \rho_{a} LC_{E} W^{\prime}\left( {\overline{{Q_{s} }} - \overline{{Q_{a} }} } \right) + \rho_{a} C_{p} C_{H} W^{\prime}\left( {\overline{{T_{s} }} - \overline{{T_{a} }} } \right)$ is a contribution of scalar wind change, $DEV = \rho_{a} LC_{E} \left[ {W^{\prime}\left( {Q_{s }^{^{\prime}} - Q_{a }^{^{\prime}} } \right) - \overline{{W^{\prime}\left( {Q_{s }^{^{\prime}} - Q_{a }^{^{\prime}} } \right)}} } \right] + \rho_{a} C_{p} C_{H} \left[ {W^{\prime}\left( {T_{s }^{^{\prime}} - T_{a }^{^{\prime}} } \right) - \overline{{W^{\prime}\left( {T_{s }^{^{\prime}} - T_{a }^{^{\prime}} } \right)}} } \right]$ is a contribution from correlated deviation terms. Using the Eq. (4), each contribution term to *THF’* was calculated using the means and deviations of each variable. We should note that bulk transfer coefficients, *C*_*E*_ and *C*_*H*_ are winds and stability dependent. To simplify the calculation and interpretation, however, the contribution of these dependencies were ignored in this study by setting *C*_*E*_ and *C*_*H*_ to 1.4 × 10^3^ and 1.5 × 10^3^, respectively. The last error term in (4) may include the error due to this simplification and defined as *Error* = *THF’ − (O* +  *A*+ *W* + *DEV).* As shown in Fig. 6, the contribution of this term is relatively small.

## Supplementary Information

Supplementary Information.

## Data Availability

The KEO and JKEO buoys datasets analysed during the current study are available in the NOAA PMEL (https://www.pmel.noaa.gov/ocs/) and JAMSTEC (http://www.jamstec.go.jp/iorgc/ocorp/ktsfg/data/jkeo/index.html). J-OFURO3 satellite-derived air-sea heat flux dataset is available in the Nagoya University (https://j-ofuro.isee.nagoya-u.ac.jp). NCEP Reanalysis dataset is provided by the NOAA/OAR/ESRL PSL, Boulder, Colorado, USA (https://psl.noaa.gov/).
