# Supplementary material for: Asymmetric air-sea heat flux response and ocean impact to synoptic-scale atmospheric disturbances observed at JKEO and KEO buoys
Source: Sci Rep. 2021 Jan 11;11:469. doi: 10.1038/s41598-020-80665-8 (PMC7801529; doi:10.1038/s41598-020-80665-8)
Supplement: Supplementary file 1 — Supplementary Information. [file 41598_2020_80665_MOESM1_ESM.docx]

**Asymmetric air-sea heat flux response and ocean impact to synoptic-scale atmospheric disturbances observed at JKEO and KEO buoys**

Hiroyuki Tomita^1^*, Meghan F. Cronin^2^, and Shun Ohishi^3^

1 Institute for Space-Earth Environmental Research (ISEE), Nagoya University, Nagoya, 464-8601, JAPAN

* tomita@isee.nagoya-u.ac.jp

2 NOAA Pacific Marine Environmental Laboratory, Seattle WA USA

3 Data Assimilation Research Team, RIKEN Center for Computational Science, Japan

Submitting to *Scientific Reports on* 2020.11.27

# Supplementary Information


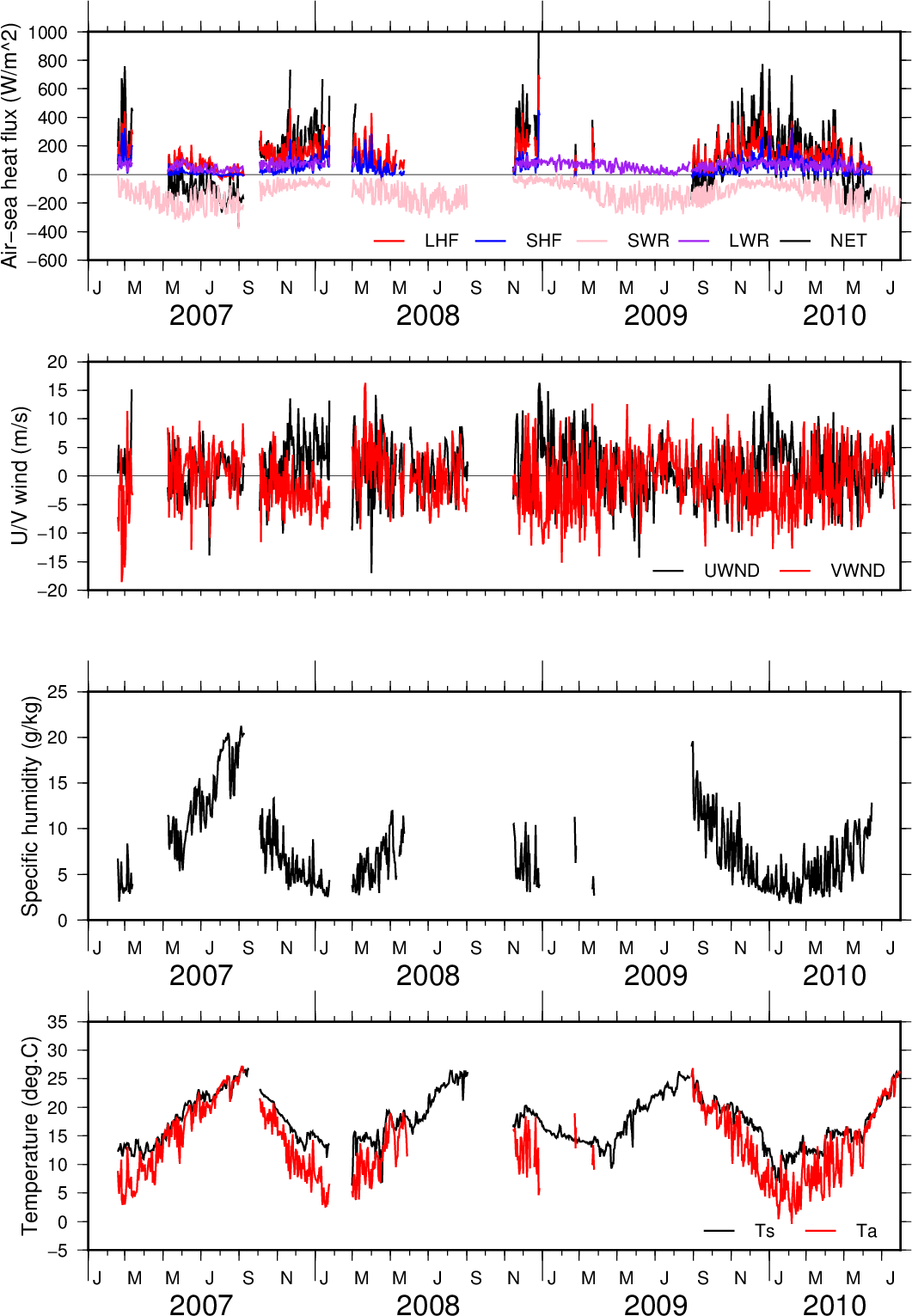


**Supplementary Figure S1:** Daily time series of surface heat fluxes and related physical parameters observed at JKEO during the full observation period from 18 January 2007 to 31 July 2010.

**
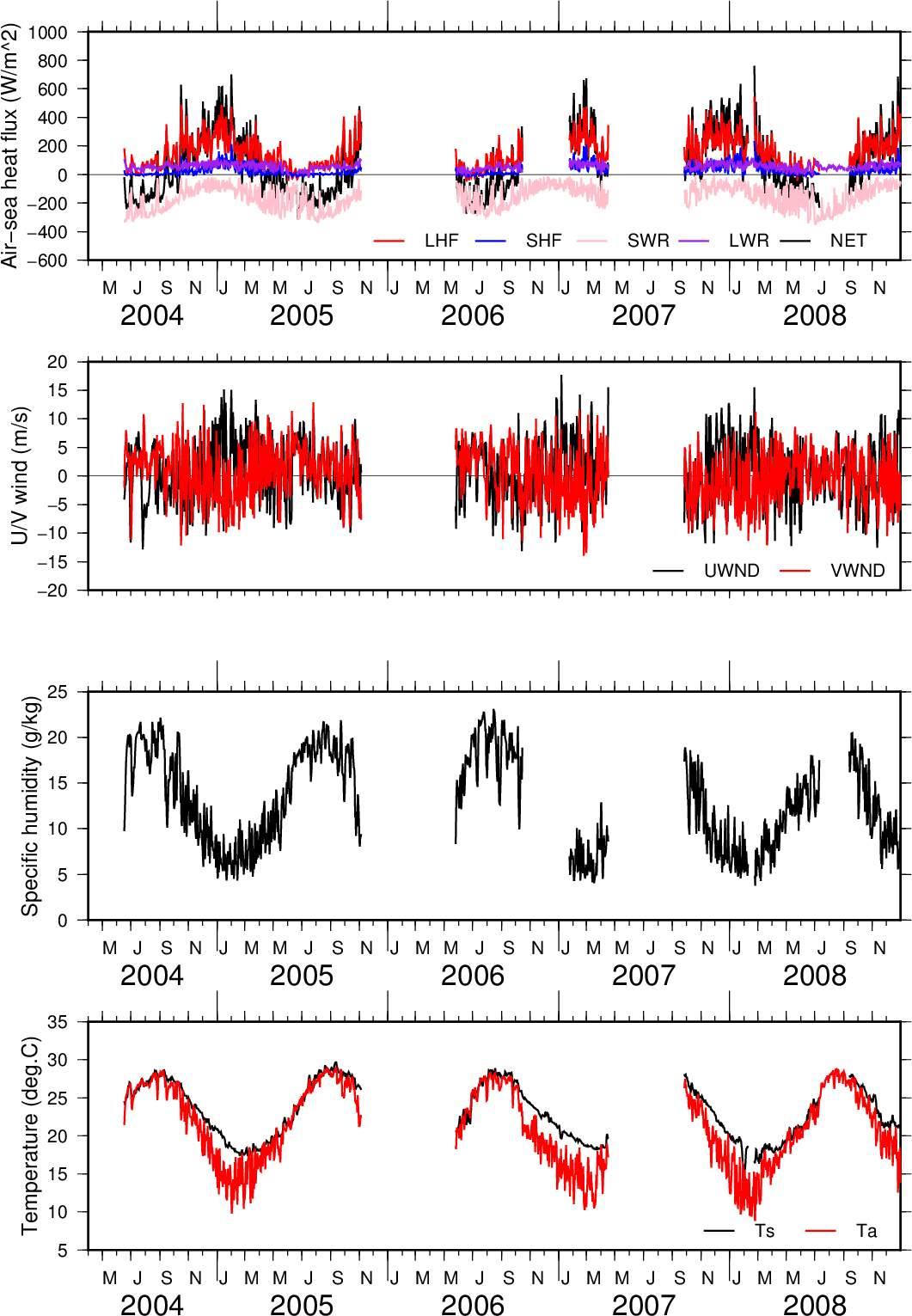
**

**Supplementary Figure S2:** Same as Supplementary Fig. S1 but for at KEO during the full observation period (a) from June 2004 to December 2008, (b) from January 2009 to December 2014, and (c) from January 2015 to December 2019.


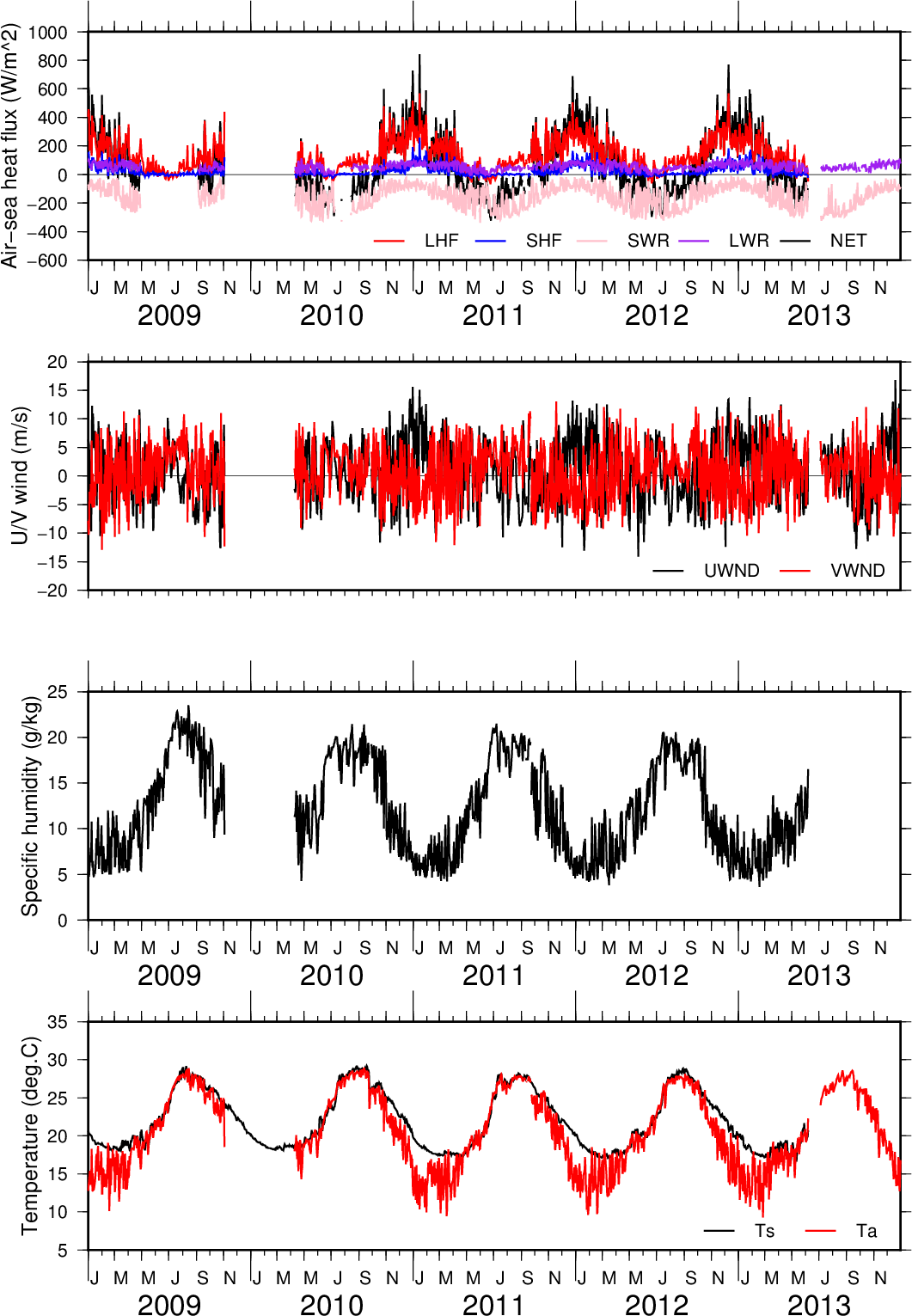


**Supplementary Figure S2 (b)**


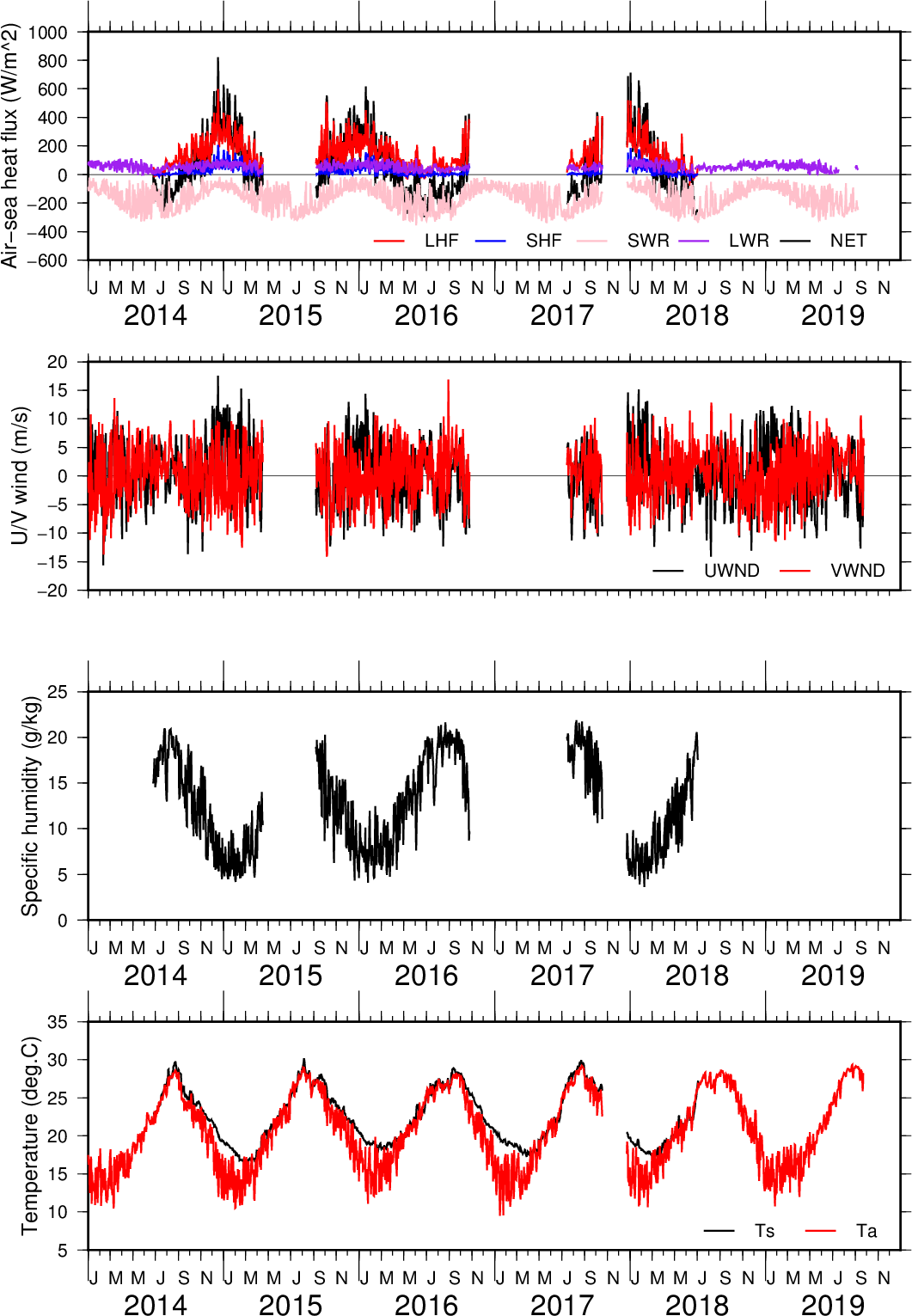


**Supplementary Figure S2 (c)**


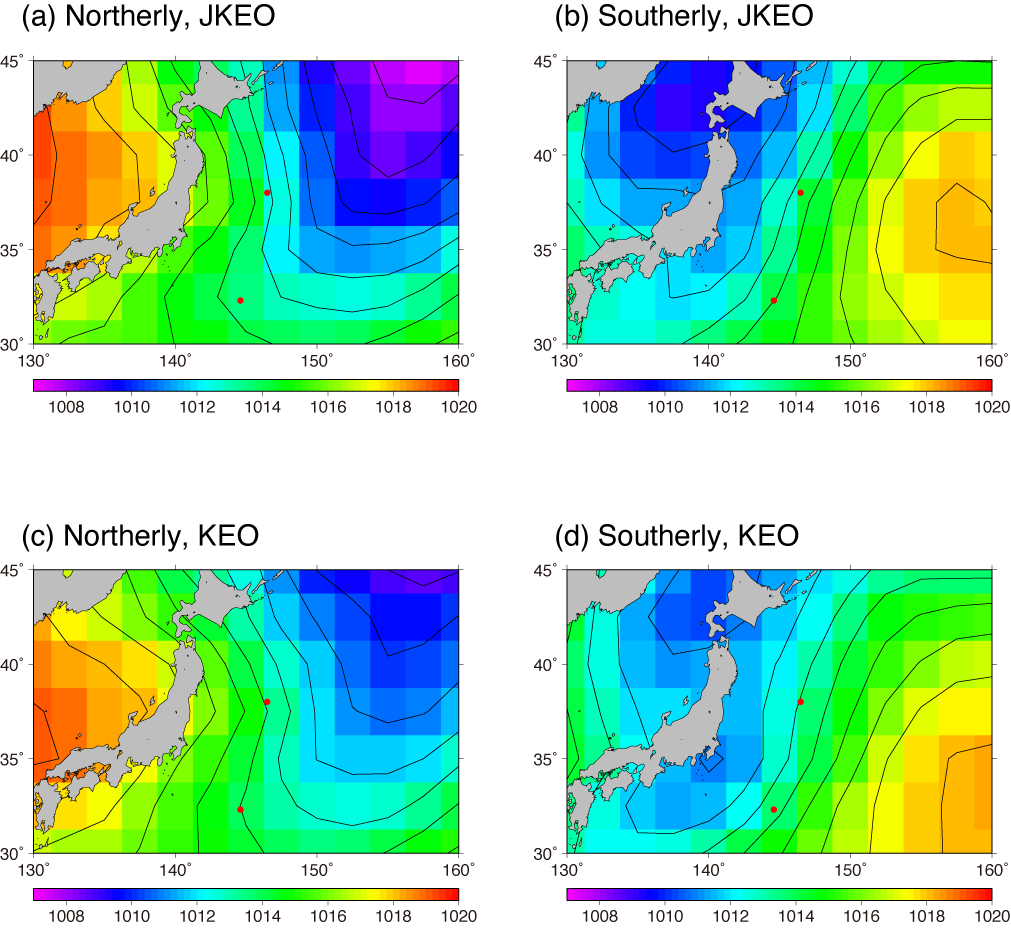


**Supplementary Figure S3:** Spatial distributions of sea level pressure (hPa) corresponding to the (a and c) northerly and (b and d) southerly wind composites at (a and b) JKEO and (c and d) KEO. JKEO (northern) and KEO (southern) buoy locations are indicated as red circles.


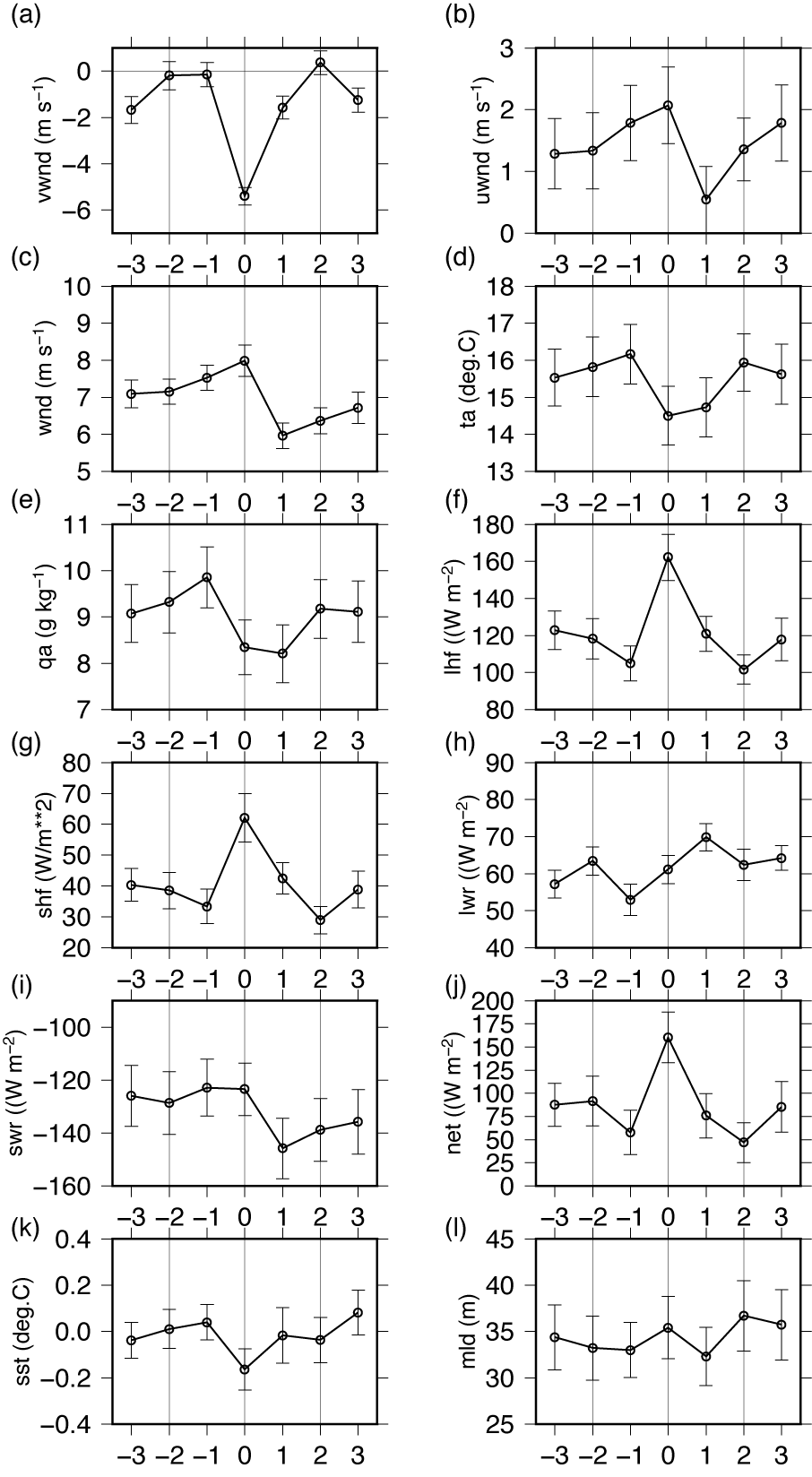


**Supplementary Figure S4:** Northerly wind composite time series at JKEO: (a) surface meridional winds (vwnd), (b) zonal winds (uwnd), (c) surface scalar winds (wnd), (d) air temperature (ta), (e) surface specific humidity (qa), (f) latent heat flux (lhf), (g) sensible heat flux (shf), (h) longwave radiation (lwr), (i) shortwave radiation (swr), (j) net heat flux, (k) sea surface temperature, and (l) mixed layer depth. The horizonal axis indicates the relative day from peak (0). The error bar indicates standard error.


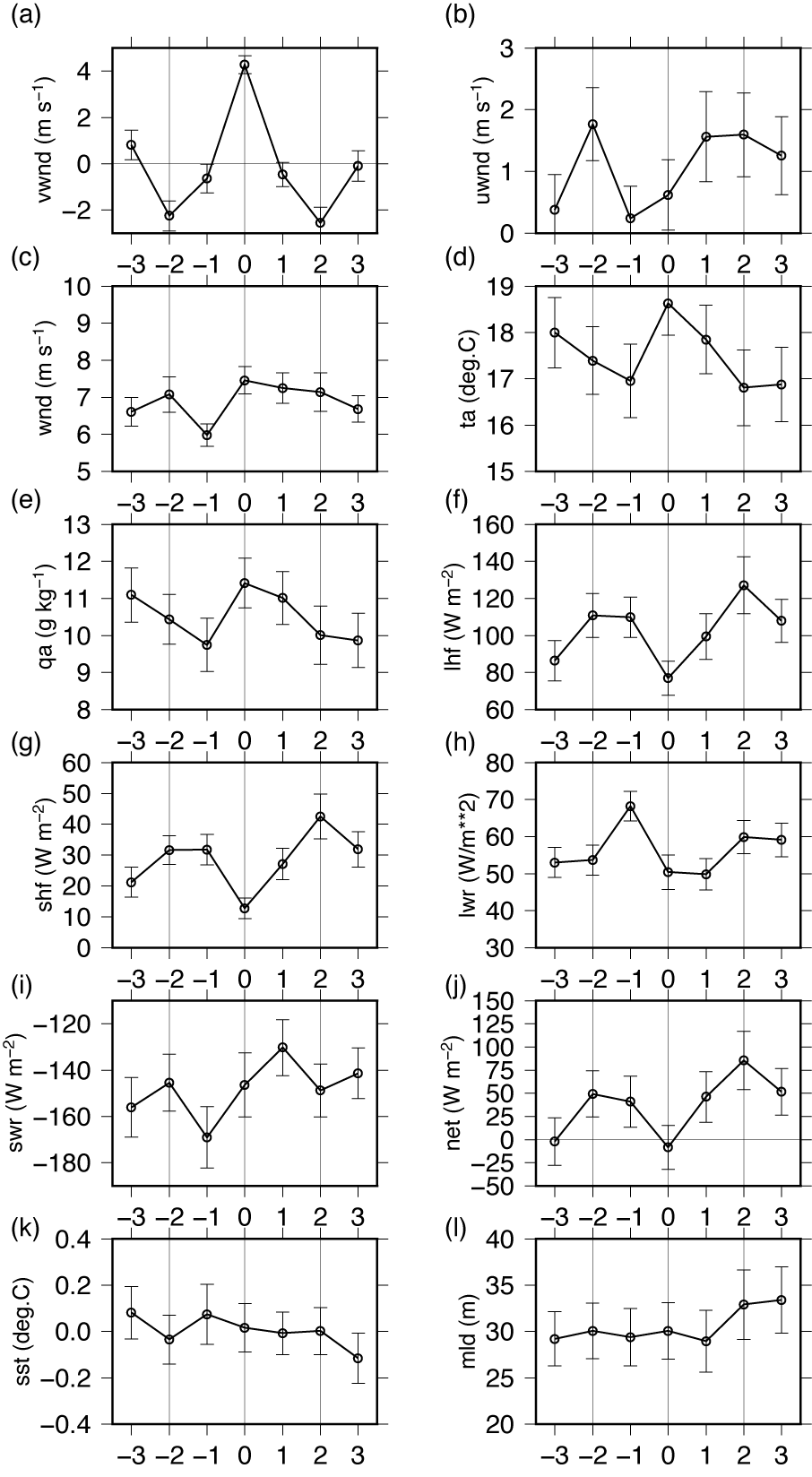


**Supplementary Figure S5:** Same as Supplementary Fig. S4, but for the southerly wind composite.


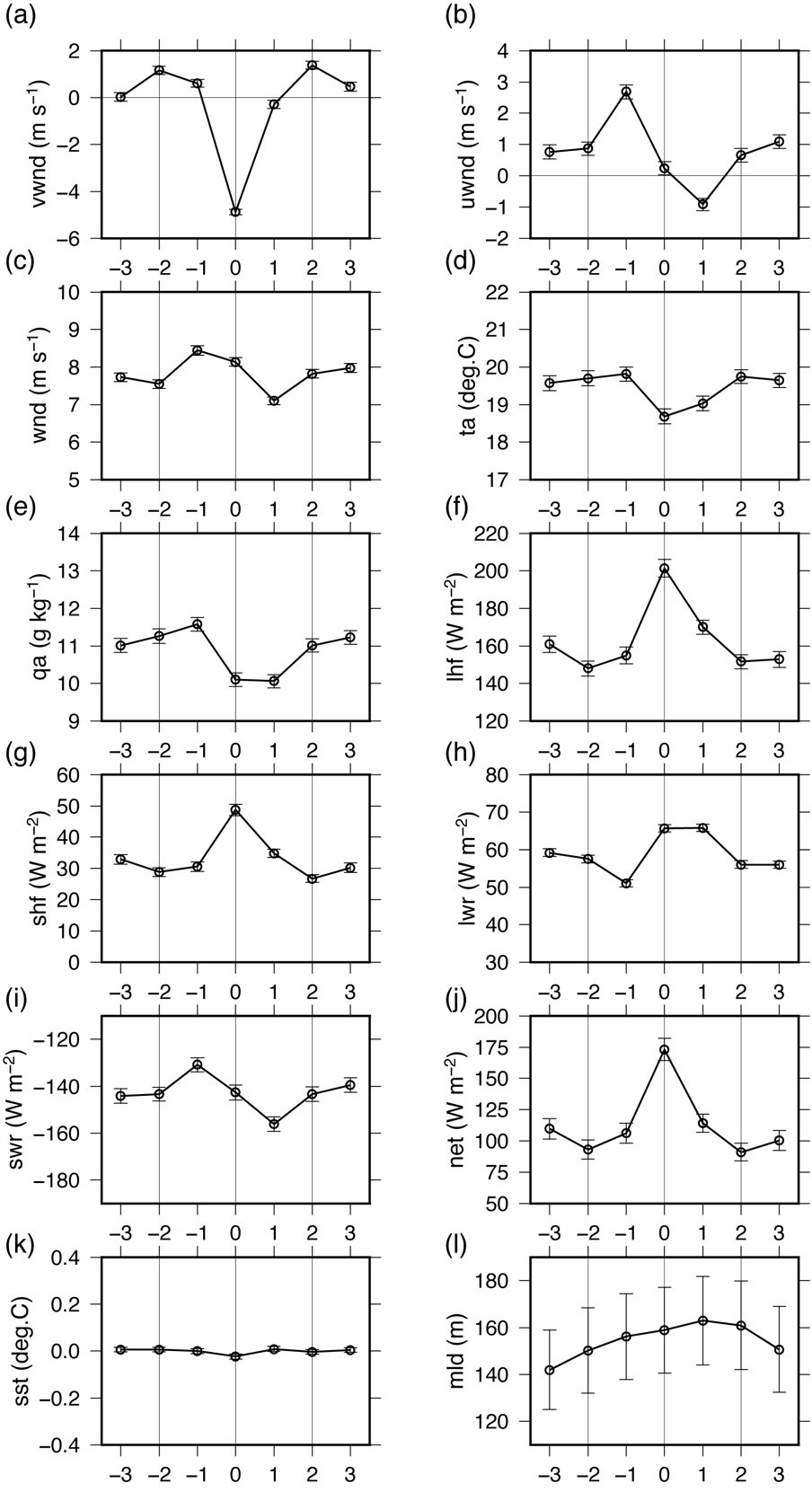


**Supplementary Figure S6:** Same as Supplementary Fig. S4, but for the northerly wind composite at KEO.


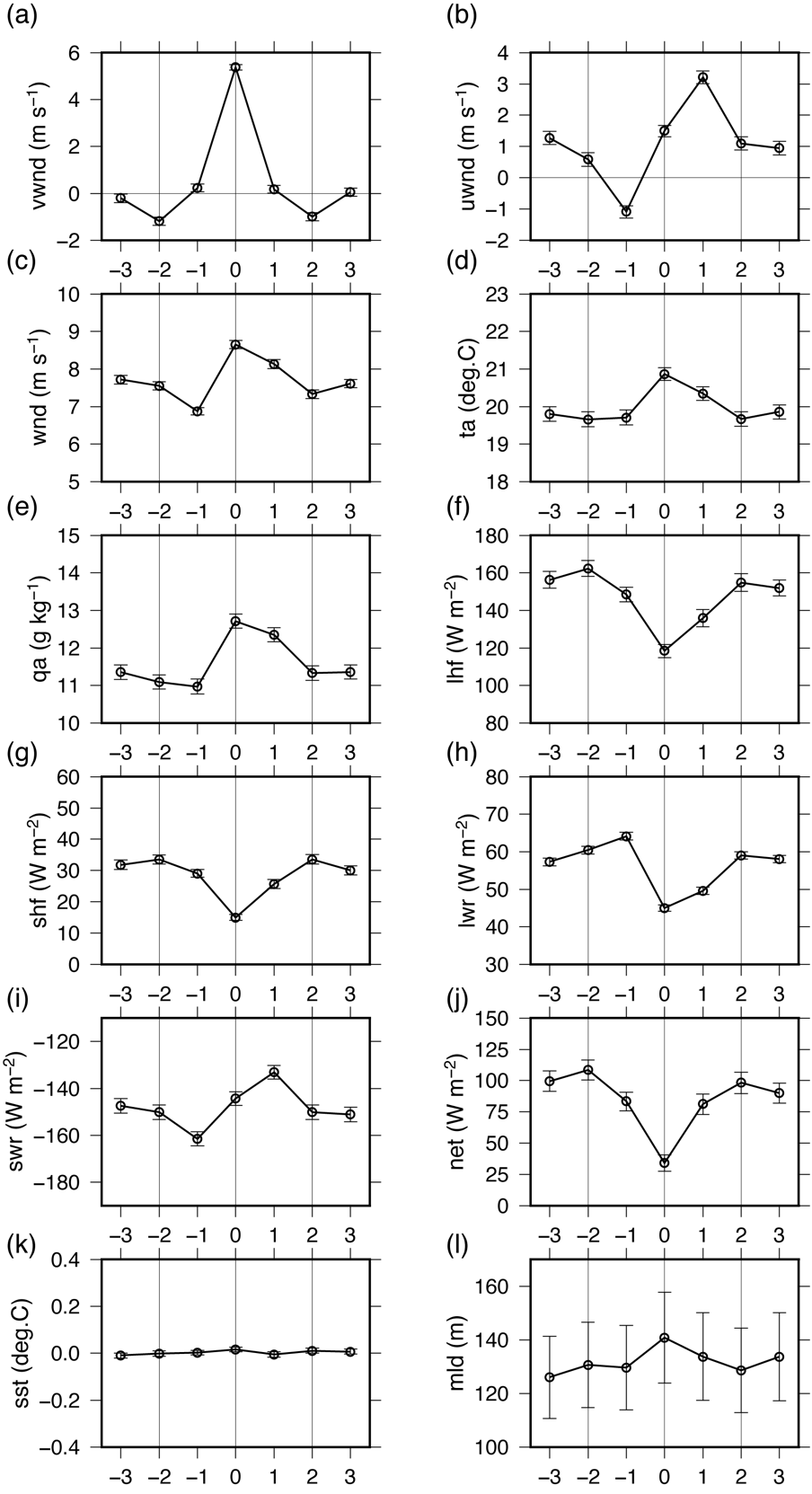


**Supplementary Figure S7:** Same as Supplementary Fig. S4, but for the southerly wind composite at KEO.


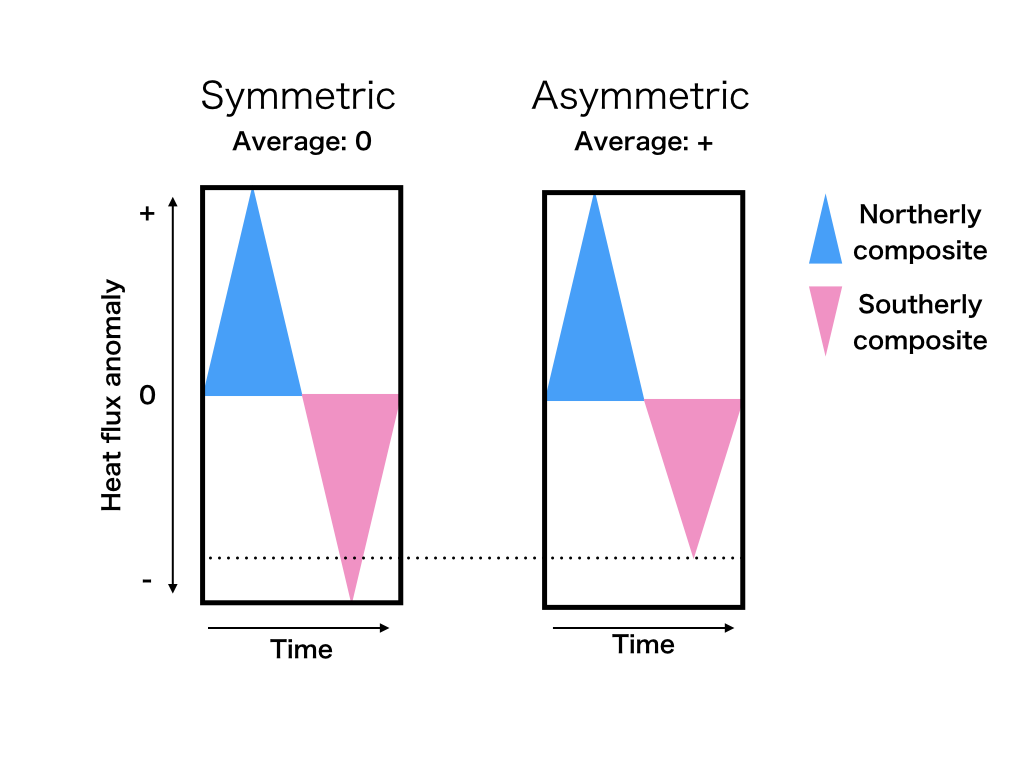


**Supplementary Figure S8:** Schematic figure of asymmetric surface heat flux responses. The two types of time series show symmetric (left) and asymmetric (right) surface heat flux responses. Blue and red indicate changes in surface heat fluxes for northerly and southerly wind events, respectively. Note that the changes associated with northerly wind events are slightly larger than those associated with southerly wind events in the asymmetric response. When time averaged, it is 0 for symmetric responses but + for asymmetric responses.


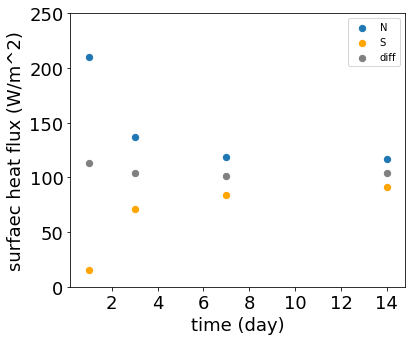


**Supplementary Figure S9:** The changes in peak value of surface net heat flux as a function of time-average scale (day). N and S are showing results for the northerly and southerly wind events at JKEO. The “diff” means the difference between N and S as an indicator of the asymmetric feature.

**Supplementary Table S1**. Summary of observations

| Buoy site name | Nominal location | Data period | Number of valid data days | Number of peaks | |
| --- | --- | --- | --- | --- | --- |
|  |  |  |  | Northerly wind | Southerly wind |
| JKEO | 38.0 ºN, 146.5 ºE | 18/02/07–15/06/10 | 542 | 125 | 108 |
| KEO | 32.3 ºN, 144.6 ºE | 16/06/04–25/09/19 | 3324 | 613 | 632 |

**Supplementary Table S2.** Statistics from observed variables and calculated surface heat fluxes at each buoy. Ave. and S.D. mean average and standard deviation, respectively. These statistics were calculated for the period described in Supplementary Table S1. U, V, TA, SST, QA, LHF, SHF, SWR, LWR, NET are zonal component of winds, meridional component of winds, surface air temperature, sea surface temperature, specific humidity, latent heat flux, sensible heat flux, shortwave radiation, longwave radiation, and net heat flux, respectively.

| Buoy | Statistics | U (m/s) | V (m/s) | TA (ºC) | SST (ºC) | QA (g/kg) | LHF (W/m^2^) | SHF (W/m^2^) | SWR (W/m^2^) | LWR (W/m^2^) | NET (W/m^2^) |
| --- | --- | --- | --- | --- | --- | --- | --- | --- | --- | --- | --- |
| JKEO | Ave. | 1.0 | -1.3 | 13.7 | 17.2 | 8.1 | 125.4 | 48.6 | -130.9 | 62.2 | 105.3 |
|  | S.D. | 4.5 | 4.9 | 6.5 | 4.3 | 4.6 | 89.4 | 57.8 | 78.9 | 28.7 | 204.6 |
| KEO | Ave. | 0.8 | 0.4 | 20.6 | 22.4 | 12.2 | 142.6 | 27.5 | -157.7 | 54.9 | 67.3 |
|  | S.D. | 5.3 | 4.6 | 5.0 | 3.8 | 5.1 | 109.1 | 36.5 | 80.1 | 25.3 | 205.3 |

**Supplementary Table S3.** Seasonal dependence of surface net heat flux at JKEO and KEO. Seasons are defined as the four different periods of December, January and February (DJF), March, April and May (MAM), June, July and August (JJA) and September, October and November (SON). The peak value corresponds to the surface net heat flux value at the peak obtained from northerly/southerly composite time series. The changes were defined as difference between the peak value and averages for the previous three days. Bold values are statistically significant changes with P-value from the t-test with 99% confidence level.

|  |  | **Northerly wind event**  **(net heat flux, W/m^2^)** | | |  | **Southerly wind event**  **(net heat flux, W/m^2^)** | | |  |
| --- | --- | --- | --- | --- | --- | --- | --- | --- | --- |
| **Buoy** | **Season** | **Peak value** | **Changes** | **N** | **P-value** | **Peak value** | **Changes** | **N** | **P-value** |
| JKEO | DJF | 372.4 | **121.0** | 38 | **0.00004** | 146.9 | **-99.1** | 22 | **0.0036** |
|  | MAM | 147.3 | **97.0** | 26 | **0.00621** | -18.0 | **-90.5** | 21 | **0.0045** |
|  | JJA | -43.3 | 54.2 | 16 | 0.08619 | -132.8 | -23.7 | 24 | 0.3931 |
|  | SON | 249.9 | **109.4** | 33 | **0.00263** | 87.7 | **-73.5** | 29 | **0.0092** |
|  |  |  |  |  |  |  |  |  |  |
| KEO | DJF | 382.0 | **91.8** | 186 | **<0.00001** | 203.5 | **-93.8** | 169 | **<0.0001** |
|  | MAM | 73.9 | **58.6** | 172 | **0.00001** | -28.1 | **-53.1** | 177 | **<0.0001** |
|  | JJA | -110.2 | 8.3 | 84 | 0.43719 | -136.4 | -7.8 | 108 | 0.4123 |
|  | SON | 180.2 | **90.2** | 164 | **<0.00001** | 39.1 | **-72.2** | 169 | **<0.0001** |

**Supplementary Table S4.** Seasonal dependence of oceanic response as sea surface temperature changes at JKEO and KEO. Seasons are defined as the four different periods of December, January and February (DJF), March, April and May (MAM), June, July and August (JJA) and September, October and November (SON). The changes were defined as difference between the peak value and averages for the previous three days. Bold values are statistically significant changes with P-value from the t-test with 95% confidence level.

|  |  | **Northerly wind event**  **(sea surface temperature, K)** | | | **Southerly wind event**  **(sea surface temperature, K)** | | |
| --- | --- | --- | --- | --- | --- | --- | --- |
| **Buoy** | **Season** | **Changes** | **N** | **P-value** | **Changes** | **N** | **P-value** |
| JKEO | DJF | 0.07 | 38 | 0.66684 | -0.31 | 22 | 0.07929 |
|  | MAM | **-0.28** | 26 | **0.02791** | 0.20 | 21 | 0.24298 |
|  | JJA | **-0.46** | 16 | **0.04782** | 0.10 | 24 | 0.67755 |
|  | SON | -0.01 | 33 | 0.95135 | -0.80 | 29 | 0.42822 |
|  |  |  |  |  |  |  |  |
| KEO | DJF | 0.01 | 186 | 0.67027 | 0.00 | 169 | 0.39486 |
|  | MAM | -0.03 | 172 | 0.25709 | 0.03 | 177 | 0.13315 |
|  | JJA | -0.08 | 84 | 0.12309 | -0.04 | 108 | 0.28929 |
|  | SON | -0.04 | 164 | 0.05460 | **0.04** | 169 | **0.02571** |

**Supplementary Table S5.** Correlation coefficients between meridional wind components and surface parameters: sea surface temperature (SST) and surface net heat flux (NET). Seasons are defined as the four different periods of December, January and February (DJF), March, April and May (MAM), June, July and August (JJA) and September, October and November (SON). Bold values are statistically significant correlation with p-values (< 0.01) from the t-test.

|  |  | **Northerly wind event** | | **Southerly wind event** | |
| --- | --- | --- | --- | --- | --- |
| **Buoy** | **Season** | **SST** | **NET** | **SST** | **NET** |
| JKEO | DJF | -0.03 | **-0.60** | -0.05 | **-0.58** |
|  | MAM | **0.19** | **-0.66** | **0.29** | **-0.62** |
|  | JJA | **0.35** | **-0.43** | **0.33** | **-0.46** |
|  | SON | 0.02 | **-0.60** | 0.09 | **-0.56** |
|  |  |  |  |  |  |
| KEO | DJF | 0.07 | **-0.60** | 0.05 | **-0.57** |
|  | MAM | **0.08** | **-0.46** | **0.08** | **-0.44** |
|  | JJA | 0.00 | **-0.13** | -0.07 | **-0.16** |
|  | SON | **0.07** | **-0.51** | **0.11** | **-0.53** |

**Supplementary Table S6.** The comparison of averaged values of net heat flux: the column “Asymmetric” indicates the values obtained from the original time series, which includes the observed asymmetric response and the column “Symmetric” indicates the averaged values obtained from the time series that has artificially removed the asymmetric features.

|  |  | Asymmetric (observation) | | Symmetric (artificially modified) | |  |
| --- | --- | --- | --- | --- | --- | --- |
| Buoy | Target | Average (W m^-2^) | Anomaly (W m^-2^) | Average (W m^-2^) | Anomaly (W m^-2^) | N |
| JKEO | All days | 105.3 | - | 88.3 | - | 542 |
|  | Northerly peak days | 230.0 | 124.8 | 154.1 | 65.8 | 121 |
|  | Southerly peak days | 21.8 | -83.5 | 21.8 | -66.5 | 101 |
|  | Other days | 84.4 | -20.8 | 84.4 | -3.9 | 320 |
|  |  |  |  |  |  |  |
| KEO | All days | 67.3 | - | 47.0 | - | 3324 |
|  | Northerly peak days | 169.8 | 102.4 | 59.4 | 12.5 | 613 |
|  | Southerly peak days | 31.5 | -35.8 | 31.5 | -15.5 | 632 |
|  | Other days | 48.0 | -19.3 | 48.0 | 1.0 | 2079 |
